# Microemulsions and Nanoemulsions in Skin Drug Delivery

**DOI:** 10.3390/bioengineering9040158

**Published:** 2022-04-05

**Authors:** Eliana B. Souto, Amanda Cano, Carlos Martins-Gomes, Tiago E. Coutinho, Aleksandra Zielińska, Amélia M. Silva

**Affiliations:** 1 Department of Pharmaceutical Technology, Faculty of Pharmacy, University of Porto, Rua de Jorge Viterbo Ferreira, 228, 4050-313 Porto, Portugal; 2 REQUIMTE/UCIBIO, Faculty of Pharmacy, University of Porto, R. Jorge Viterbo Ferreira, 228, 4050-313 Porto, Portugal; 3 Department of Pharmacy, Pharmaceutical Technology and Physical Chemistry, Faculty of Pharmacy, University of Barcelona, 08007 Barcelona, Spain; acanofernandez@ub.edu; 4 Institute of Nanoscience and Nanotechnology (IN2UB), University of Barcelona, 08007 Barcelona, Spain; 5 Centre for Research and Technology of Agro-Environmental and Biological Sciences, CITAB, UTAD, Quinta de Prados, 5001-801 Vila Real, Portugal; camgomes@utad.pt (C.M.-G.); tcoutinho4@hotmail.com (T.E.C.); 6 Department of Biology and Environment, University of Trás-os-Montes e Alto Douro, UTAD, Quinta de Prados, 5001-801 Vila Real, Portugal; 7 Institute of Human Genetics, Polish Academy of Sciences, Strzeszyńska 32, 60-479 Poznań, Poland; aleksandra.zielinska@igcz.poznan.pl

**Keywords:** microemulsions, nanoemulsions, skin drug delivery, skin bioavailability

## Abstract

Microemulsions and nanoemulsions are lipid-based pharmaceutical systems with a high potential to increase the permeation of drugs through the skin. Although being isotropic dispersions of two nonmiscible liquids (oil and water), significant differences are encountered between microemulsions and nanoemulsions. Microemulsions are thermodynamically stable o/w emulsions of mean droplet size approximately 100–400 nm, whereas nanoemulsions are thermodynamically unstable o/w emulsions of mean droplet size approximately 1 to 100 nm. Their inner oil phase allows the solubilization of lipophilic drugs, achieving high encapsulation rates, which are instrumental for drug delivery. In this review, the importance of these systems, the key differences regarding their composition and production processes are discussed. While most of the micro/nanoemulsions on the market are held by the cosmetic industry to enhance the activity of drugs used in skincare products, the development of novel pharmaceutical formulations designed for the topical, dermal and transdermal administration of therapeutic drugs is being considered. The delivery of poorly water-soluble molecules through the skin has shown some advantages over the oral route, since drugs escape from first-pass metabolism; particularly for the treatment of cutaneous diseases, topical delivery should be the preferential route in order to reduce the number of drugs used and potential side-effects, while directing the drugs to the site of action. Thus, nanoemulsions and microemulsions represent versatile options for the delivery of drugs through lipophilic barriers, and many synthetic and natural compounds have been formulated using these delivery systems, aiming to improve stability, delivery and bioactivity. Detailed information is provided concerning the most relevant recent scientific publications reporting the potential of these delivery systems to increase the skin permeability of drugs with anti-inflammatory, sun-protection, anticarcinogenic and/or wound-healing activities. The main marketed skincare products using emulsion-based systems are also presented and discussed.

## 1. Introduction

Transdermal delivery is a convenient form of drug administration, as it allows a controlled release of the drug with minimum side effects and a higher patient compliance rate. Compared to the oral route, transdermal administration offers the additional advantage of overcoming the first-pass metabolism, also avoiding the gastrointestinal side effects (e.g., diarrhea, nauseas or even drug degradation in the gut) [1]. Hydrophilic drugs, however, cannot easily pass through the skin because of the presence of the *Stratum corneum*. This outermost layer of the skin presents an accumulation of flattened and anucleated keratinocytes (known as corneocytes) that represent an important physical barrier against the entrance of xenobiotics; however, also preventing the delivery of therapeutic drugs. The permeation of drugs through the skin is the limiting factor contributing to their bioavailability via the transdermal route, and is governed by their *p*Ka and oil-in-water partition [2,3,4].

**Figure 1:**
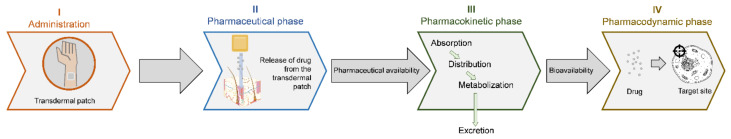
From drug administration to its pharmacological effect. Upon administration (phase I), the drug is released from the pharmaceutical patch through the skin (phase II), after which the components are pharmaceutically available to be processed by the phase III-ADME system (absorption, distribution, metabolism and excretion). Once the drug reaches the systemic circulation, it becomes bioavailable to proceed to the target site and perform the pharmaceutical action (phase IV).

To reach the target site, the administered drug undergoes a pharmaceutical phase (i.e., characterized by its release from the dosage form), a pharmacokinetic phase (i.e., undergoing absorption, distribution, metabolization and, eventually, a portion of it is excreted from the body before reaching the target site) and a pharmacodynamic phase (when reaching the systemic circulation and the site of action) (Figure 1) [5].

As can be seen in Figure 1, the action of a drug after its administration undergoes a set of processes described in three phases, namely, the pharmaceutical phase, the pharmacokinetic phase and the pharmacodynamic phase [5]. The pharmaceutical phase is characterized by the moment when the vehicle releases the drug into the skin [5]. After its dissolution, the drug undergoes a set of pharmacokinetic reactions that include absorption, body distribution, metabolism by organs and elimination by excretion systems (ADME: absorption, distribution, metabolism and excretion). After drug release, the drug is available to interact with its target (i.e., receptor and enzymes) in order to produce the therapeutic effect, that is, the pharmacodynamic phase [5,6]. All of these processes and steps show that the action of drugs depends not only on the nature of the molecule, but also on the carrier itself and on the target site [7,8].

Over the last two decades, remarkable advances have been observed in a new technology, standing out due to working with particles smaller than 1 µm, namely, nanotechnology [9,10,11,12]. This multidisciplinary field of study also covers the health science area. The production of nanoparticles, whose scale is similar to that of cellular organelles, allows an adequate transport of small molecules to target cells, reducing the waste of the drug and the administration dose, while at the same time increasing its therapeutic efficacy. Although a newcomer to the market, there is already some evidence that demonstrates the enormous potential of nanoparticles in improving the therapeutic activity of drugs, both in their action and in their application through different routes of administration according to the convenience and need of the patient [13,14,15].

Among the various formulations in the field of nanotechnology, nanoemulsion represents a versatile option for the delivery of drugs through lipophilic barriers. Currently, this technology is already under use in several industries, as is the case in food, biomedical and cosmetic industries [16]. Nevertheless, the development of nanoemulsions for skin delivery of either cosmetics or therapeutic drugs has arisen as a strategy to overcome some limitations, such as the penetration and absorption of these active molecules.

In this review, we discuss the application of emulsions as efficient delivery systems for the transportation of drugs through skin, as well as the advantages of micro- and nano-sized versions of this technology. We address recent developments in microemulsions and nanoemulsions developed for both pharmaceutical products (e.g., anti-inflammatory drugs) and the cosmetics industry (e.g., antiaging drugs), describing the wide variety of natural and synthetic lipids used, the resulting physical and chemical properties and the main findings obtained in various in vitro, in vivo and ex vivo experimental models.

## 2. Pharmaceutical Emulsions

Formulations based on dispersions of solid particles, polymers or liquid droplets compose part of the therapeutic systems. Their composition is based on a continuous viscous phase, where another different phase is dispersed, and results in small dispersed particles, not noticeable to the naked eye, which is why they are often called colloids [17]. This group includes the large family of emulsions, whose droplet dimensions can vary between 100 nm and 100 µm, depending on their nature [18,19].

Commonly, in the emulsion family, the hydrophilic phase consists of water, and the lipophilic phase is composed of one or more lipids that are liquid at room temperature. These are nonpolar compounds of a similar structure to those present in the body and in food products, and, thus, with increased biocompatibility [20]. However, for each formulation, it is necessary to take into account many factors, i.e., the nature of the system and the equilibrium point of the different constituents. The proportion of the phases that compose the emulsions is crucial for the behavior of these formulations, being a very important aspect. All of these characteristics can be decisive of parameters, such as the viscosity and visual appearance of the emulsion [17]. As the particle size decreases, the emulsion color changes from milky white to translucent. In cases where the droplets have dimensions greater than 1 µm, the emulsion has a milky white tint due to the scattering of light, a consequence of the different refractive indices of the internal and external phases [18].

**Figure 2:**
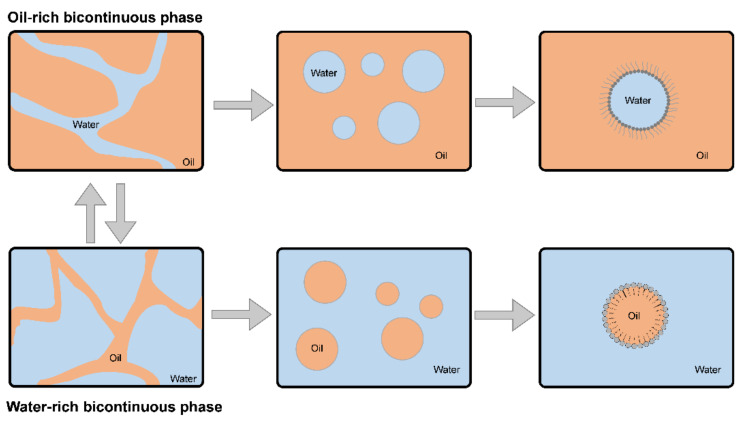
Representation of colloidal dispersions of o/w and w/o types.

The nature of the continuous and discontinuous phases also influences the nature of each colloidal system. A colloidal dispersion can be water/oil (w/o) or oil/water (o/w) [21], as shown in Figure 2 [22].

In general, emulsions are capable of solubilizing substances of polar and nonpolar natures. This is due to the organization of the oil and water phases [21]. The most frequent emulsions applied in pharmaceutics are o/w, which allow the transport of a lipophilic substance in the internal phase of the emulsion [21,23]. Additionally, o/w emulsions are the most appropriate for topical application, as they allow the penetration of hydrophilic substances through the *Stratum corneum* of the skin. With some exceptions, these systems are not produced spontaneously due to their thermodynamic instability. Usually, the preparation of an emulsion is carried out by mechanical stirring to homogenize the two heterogeneous phases. However, when the external force on the o/w mixture ceases, there is a break in the balance of the emulsion, which can lead to the degradation of its characteristics over time, due to the tendency of separation between phases [17,18,21,24,25,26]. This trend is the result of the difference between the forces of attraction that occurs between the molecules of the two liquid phases, called interphase tension [17]. Interactions of this nature can be enumerated [27], namely: (i) when the particle collision is stable, a film is formed, with the possibility of flocculation due to the existence of aggregated particles; (ii) when the force of attraction between droplets is predominant, the film is unstable and breaks, which leads to coalescence or foaming phenomena; (iii) if repulsion forces are prevalent, the colliding pair of particles repel and prevent particle aggregation and consequent phase separation, making the system more stable [19]. Thus, in general, emulsions exhibit metastable colloid behavior, exhibiting flocculation, creaming and separation phenomena as a result of droplet interactions [21]. To counter this type of phenomenon, it is necessary to maintain the stability of the emulsion. In this sense, a number of mechanisms are available, such as electrostatic, steric stabilization or stabilization by solid particles [17,24]. However, the stability of most pharmaceutical formulations is guaranteed by the addition of surfactants [21,24].

**Figure 3:**
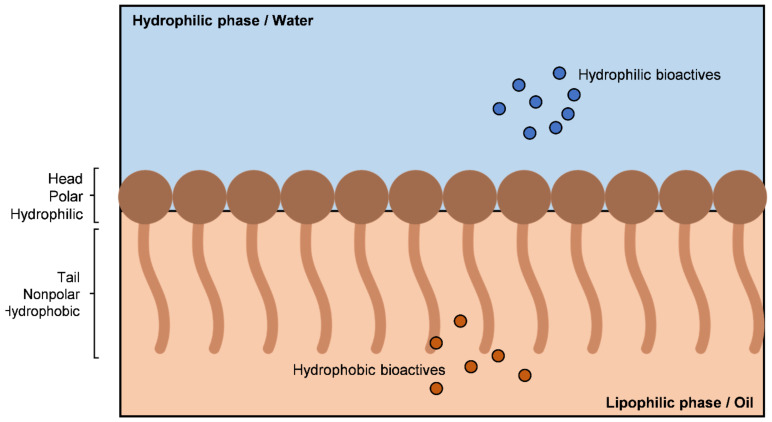
Schematic representation of surfactant placement in the oil/water interface.

The amphiphilic nature of surfactants induces a reduction in the interphase voltage of the systems. As can be seen from Figure 3, the structure of the surfactants causes them to be adsorbed at the interface of the mixture, as they have a lipophilic “tail” and a hydrophilic “head” [17].

Two types of surfactants can be considered, the primary and the secondary. The primary surfactants are those whose properties allow the stabilization of the emulsions without the need to add any other component for this purpose [24]. Secondary surfactants, or cosurfactants, have polar and nonpolar structures in the same molecule (amphiphilic characteristics) and serve to increase the thermodynamic stability of these systems [24].

In order to ensure the long-term stability of the emulsion, avoiding or postponing phenomena such as coalescence, an appropriate choice of surfactants is important. These, in general, must be more soluble in the external phase of the system than in the internal phase [17]. The classification system for the tendency of a surfactant molecule to disperse in the lipophilic or hydrophilic phase is referred to as the hydrophilic–lipophilic balance [28], with a numerical classification list of surfactants in relation to their solubility in an aqueous or lipid medium [17,21].

Another way to classify surfactants is by their structure as anionic, nonionic, cationic, amphoteric or zwitterionic [21]. Surfactants have different characteristics, with their choice depending on the type of application and formulation that is intended for preparation, in order to obtain an effectively emulsified mixture [21,24].

**Figure 4:**
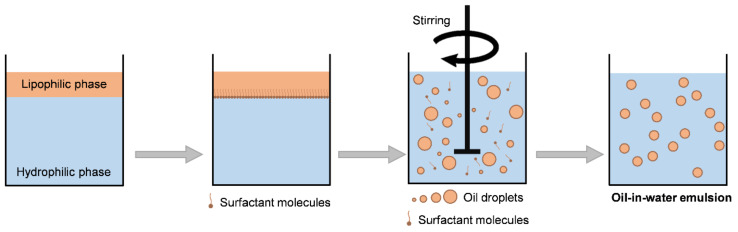
Production of an o/w emulsion.

The addition of one or more surfactants to this immiscible mixture guarantees the formation of a macroscopically homogeneous and microscopically heterogeneous phase [24]. When considering the aforementioned steps, it is possible to summarize the production of an emulsion by the scheme shown in Figure 4 [17].

Most of the emulsions follow the Bancroft rule, formulated in 1913. According to Bancroft, the type of emulsion depends more on the nature of the surfactant than on the lipophilic/hydrophilic substance ratio, or even on the preparation methodology itself. That is, the phase in which the surfactant is soluble is the external phase [21,27,29]. In general, emulsions have the purpose of making a drug more amphiphilic, regardless of its nature, in order to become more compatible with biological systems [30].

An o/w emulsion can be converted into a w/o emulsion and vice versa. This is possible by (i) changing the order of phase addition, (ii) changing the phase volume ratio, (iii) temperature variations and (iv) by the presence of electrolytes and other additives such as alcohol, leading to changes of the surfactant solubility and resulting in a phase inversion [21,31]. The inversion of the phases of an emulsion is particularly useful when the final emulsion is subject to specifications that are not supported by more conventional emulsification methods, such as when obtaining small droplets of the internal phase or very viscous oils [21,29].

Microemulsions and nanoemulsions receive special attention due to their high potential compared to other colloidal systems, namely, high optical clarity, good physical stability and the ability to improve the bioavailability of various drugs [32]. This type of emulsion has a set of characteristics, making it appropriate to group them in the area of nanotechnology; more precisely, in nanomedicine. Thus, in order to better understand the way micro and nanoemulsions work, it is important to understand the fundamentals of this new technology.

## 3. Principles of Drug Delivery

The last few decades have been auspicious for the emergence of numerous pharmaceutical system techniques, especially regarding formulations of new drug delivery systems. Nanotechnology was one of the main research ventures of the 21st century [11].

In the health science area, the drug delivery of therapeutic molecules through vehicles or formulations suitable for this purpose is not a new concept. However, formulations considered classic, as a rule, have a number of limitations, namely, (i) a high dose of drugs administered but poor bioavailability, (ii) the first-pass effect that potentially reduces the bioavailability of drugs, (iii) risk of toxic effects, (iv) instability during storage and (v) a marked plasma fluctuation of the drugs’ concentration, which hinders a sustained effect [33,34]. Thus, there is a need to find a type of system that allows the release of drugs in a more effective way and without so many side effects.

Nanotechnology is also applicable for pharmaceutical uses, offering a set of possibilities that were inaccessible until recently. Initially, nanomedicine was mainly applied to the areas of engineering and computing [35], even going so far as to state that: “*The initial concept of the perception of nanomedicine was launched as an idealistic way of thinking that tiny nano-robots, and related machinery, can be planned, manufactured, and introduced into the human body to perform cellular maintenance and repair at the molecular level*”.

Nanomedicine and nanotechnology create countless opportunities for medical and scientific advancement in the treatment of diseases in the population [36,37]. In the field of pharmaceutical sciences, operating at the nanoscale makes it possible to explore different physical properties distinct from the microscale, such as the volume/surface ratio [36]. Thus, nanoparticles have a high specific surface, which increases their ability to adhere to the site of action, increasing the time interval for the release of the substances. The prefix “nano” is derived from the Greek word “νᾶνος” (pronounced as “nanos”) meaning “dwarf” [37]; therefore, nanotechnology makes use of nanometric particles, that is, particles whose scale fits the nanodimensions [9,10].

Overall, nanotechnology can offer advantages in drug delivery that include (i) enhancing the drug bioavailability by favoring its solubility [33,38,39,40], (ii) increase in the drug’s half-life, allowing it to have enough time to interact with the target cells [33,41,42], and (iii) potential site-specific targeting [33,38,39,40].

The drug delivery of pharmacologically active compounds that are poorly soluble in aqueous media, using lipids, has been extensively investigated [38]. These types of systems have been shown to be useful in promoting the bioavailability of lipophilic drugs due to their ability to keep drugs dissolved within the system, while surpassing physiological barriers, until reaching the target site [38]. There are several types of nanoparticles composed of lipids, such as solid lipid nanoparticles, nanostructured lipid vectors, microemulsions and nanoemulsions [33], whose properties enable the solubilization and controlled release of various drugs.

## 4. Micro and Nanoemulsions

Emulsions, such as micro and nanoemulsions, are composed of droplets of small dimensions, below 1 µm; that is, on the nanometer scale. However, their physical and chemical properties determine their application in pharmaceutics [18,43]. Microemulsions are considered thermodynamically stable dispersions of mean droplet size approximately 100–400 nm, whereas nanoemulsions are thermodynamically unstable dispersions of mean droplet size approximately 1 to 100 nm that usually require a cosurfactant for their stabilization due to the higher free energy [44]. The quantitative and qualitative composition of this type of emulsion influences the average particle diameter and its polydispersity index. In fact, the relationship between the chosen lipid, which influences the viscosity, lipophilic and interphase tension between phases, and the surfactant, which reduces the free interphase energy of the system, are determining factors for the particle dimensions obtained [10]. A key factor for drug delivery is the small size and uniformity of the droplet, which vary according to the type of administration and the target cells for which it is intended [10]. The higher the polydispersity index, the larger the particle size range and, consequently, the more heterogeneous the emulsion is.

The zeta potential is another fundamental parameter for the thermodynamic stability of emulsions [45]. This translates into the electrical charge that the particles present on its surface and evaluates the capacity that the particles are able to maintain in suspension, that is, without aggregating or sediment [46]. In this way, the further away the zeta potential value is from zero in absolute value, the greater the repulsion between the suspended particles.

The very composition of colloidal dispersions dictates their behavior. Thus, it is important to establish a good quantitative relationship between the variables of the formulation in order to achieve a system with ideal properties to achieve an effective drug delivery [45]. All of these parameters can be studied and evaluated statistically in order to facilitate the study of the formulation.

The existence of large amounts of lipids in the skin allows lipid vectors to have a greater affinity for this pathway [47]. This route provides a greater lipid exchange between the layers of the skin and the vehicle, inducing it to a better penetration of the drugs. Small particles have a greater ability to adhere to the skin due to the greater surface area they present (the adhesion of a nanoparticle is inversely proportional to its size), with nanoparticles of about 200 nm being suitable for topical application [43].

There are some therapies for topical diseases (eczema, acne and psoriasis) that use corticosteroids and retinoids. However, the classic formulations do not have a high penetration capacity, which leads to the appearance of atrophy on the skin, skin irritation or higher sensitivity in the application area [28]. Nanoemulsions offer an enormous possibility to increase the penetration of drugs, which leads to an increase in its therapeutic efficacy and the consequent reduction in its side effects.

### 4.1. Microemulsions

Microemulsions have been investigated for the past four decades, especially in the late 1970s and early 1980s [48]. The definition of a microemulsion was proposed in 1943 by Hoar and Shulman [49,50]. These scientists described microemulsions as translucent systems formed spontaneously, consisting of a set of droplets limited by an interphase film, which is composed of a mixture of a surfactant and, if necessary, a cosurfactant [25,49]. They are called microemulsions due to the small size of the droplets compared to the emulsions [21]. Thus, microemulsions can be described as the mixture of water with a surfactant that, in the appropriate proportions and together with a lipophilic phase, originates droplets with diameters of less than 140 nm [18,25,51,52,53]. The droplets that make up colloidal dispersions of this nature are framed within a nanometric range, which results in the term “microemulsion” to generate some confusion as to the characteristics of these systems, as it is not the most suitable [53]. These colloids can be considered nanoreactors because they are often used to transport chemical agents, as is the case with drugs [52].

Microemulsions can be ternary or quaternary, composed of a mixture of oil, water, surfactant (ternary) and, if necessary, cosurfactant (quaternary), whose mixture can give rise to droplets that form a stable dispersion, which can vary from a milky white to a translucent formulation [25,51,53,54]. This appearance is governed by the size of the droplets that compose the microemulsions, which should not exceed a quarter of the wavelength of visible light (approximately 150 nm) [18,53]. In microemulsions, the application of a cosurfactant serves to reduce the tension of the surfactant film, which results in a more flexible and dynamic layer [53].

These systems have a high thermodynamic stability and isotropy, where the combination of the two immiscible liquids (usually oil and water) is stabilized by surfactants that are located at the o/w interface [25,49,53]. These emulsions are widely used as chemical reactors due to their special interface characteristics that establish an intimate contact between the hydrophilic and lipophilic phases, at the nanometric level [55]. These systems provide a compartmentalized, versatile and unique environment that provides different types of reactions.

Microemulsions differ from classic emulsions because of their thermodynamic stability. Thus, the mixture of adequate proportions of each of the components of the system, and under conditions of constant temperature, pressure and ionic strength, causes it to be formed spontaneously [25]. Microemulsions have some points in common with emulsions, such as the possibility of obtaining formulations of an o/w or w/o type, the relationship between the proportions of the surfactant and cosurfactant and the balance of the hydrophilic/lipophilic phases [21,25,51]. This combination of factors gives rise to a very fine emulsion, obtained by gentle stirring, with very small (less than 140 nm) droplets [51]. It is, in fact, the thermodynamic stability of microemulsions that causes them to be formed spontaneously, when conditions and components in an adequate quality and quantities are brought together [18]. In order to find the balance between the elements of the system, it is necessary to carry out a phase diagram [56,57].

The phase diagram is a tool that describes the behavior of the various components of a microemulsion [58]. As a rule, it must be built according to the type of system used, where the behaviors of combinations of different proportions of an oil, water and surfactant/cosurfactant mixture are evaluated in relation to a fixed surfactant/cosurfactant ratio. Through these tests, the points of formation of monophasic and biphasic systems are observed visually. Systems in which turbidity is observed together with phase separation are considered biphasic. On the other hand, after gentle agitation, single-phase systems are presented as clear and translucent mixtures, and are represented by dots in the phase diagram. The area covered by these points is considered to be the microemulsion region. From this information, the microemulsion is prepared, combining the proportions of the components that cover the area of the single-phase system, gradually and with the aid of gentle agitation, adding the drug in the internal phase of the formulation. After these steps, an ultrasonicator can be used to obtain droplets of suitable dimensions from the dispersed phase until equilibrium is reached. The ultrasonicator emulsifies by means of ultrasound through two processes. First, the application of an acoustic field that produces waves in the interphases causing them to become unstable results in the eruption of the oil phase in the aqueous medium in the form of droplets. Then, the application of low ultrasound frequencies causes an acoustic cavitation, that is, the formation and subsequent collapse of microbubbles that induce turbulence in the system, which is an effective method in the “breaking” of droplets, inducing the formation of a very fine dispersion [59,60,61].

Secondly, several studies on the application of microemulsions to the skin for the drug delivery of hydrophilic and lipophilic pharmacologically active compounds have shown to be quite promising [62]. A study [57] aimed to deliver penciclovir (a potent antiviral agent used to treat diseases caused by herpes simplex, varicella zoster, Epstein–Barr virus, hepatitis virus and cytomegalovirus), a drug with a very low oral bioavailability, sold in the form of a cream, for topical use. These authors chose to develop a microemulsion of penciclovir, whose action was compared with the commercial cream of this substance on the skin of mice. The result obtained showed that the microemulsion had a penetration capacity 3.5 times higher than the cream available on the market, which could represent a substantial improvement in the effectiveness of this drug [57].

### 4.2. Nanoemulsions

There is a wide variety of definitions of a nanoemulsion [20]; in a universal way, these systems can be considered colloidal systems, similar in structure to emulsions and microemulsions. This type of system involves finely dispersed droplets on a nanoscale, having great promise in the future of biotechnologies, cosmetics and drug delivery [20,63,64].

The unique droplet characteristics of these systems allow for effective drug delivery. In addition, nanoemulsions provide a number of advantages over conventional emulsions, such as: (i) weak light scattering; hence, a tendency to be transparent or translucent; (ii) high thermodynamic stability, avoiding droplet aggregation or gravitational separation; (iii) unique rheological characteristics (such as reduced viscosity); (4) being able to considerably increase the bioavailability of lipophilic compounds [65].

Visually, microemulsions and nanoemulsions are very similar. They are systems with a milky or translucent aspect due to their small particle diameter and low viscosity, where the drug conveyed is dispersed and/or adsorbed in the internal phase of the droplets [66]. Nanoemulsions are also known as miniemulsions or ultrafine emulsions [23,67], and consist of an immiscible mixture of isotropic liquids, composed of a lipophilic phase, a hydrophilic phase, surfactant and, if necessary, a cosurfactant [20,30,68,69,70,71,72]. Similar to other systems, the surfactant involves the droplet forming a film in order to stabilize the dispersed phase, counteracting the occurrence of coalescence phenomena [20,63,68,69,70].

Some authors indicate that the droplet size in a nanoemulsion must be less than 100 nm [20,30,63,64,70]. Standard nanoemulsions, which are produced by high-pressure homogenization, have difficulty in reaching droplets with dimensions within this range, which also applies to nanoemulsions, with droplet dimensions between 100 and 500 nm [20,63]. Nanoemulsions are formulations composed of a set of physiologically accepted excipients, which can be produced on a large scale and subjected to sterilization processes [73]. These are metastable systems whose characteristics and physico-chemical stability are dependent on the adopted production processes [20,64,72].

There is some disagreement regarding the thermodynamic stability of nanoemulsions. Initially, some authors suggested that, like microemulsions, nanoemulsions are systems of high thermodynamic stability [72]. However, it was later discovered that these systems have a reduced thermodynamic stability. As nanoemulsions are composed of droplets of very small dimensions, they are not affected by gravity, but are subject to Brownian motion, the same one that prevents or delays conventional destabilization phenomena, as is the case in coalescence, as it gives them a kinetic stability [20,64,69,70,74,75]. Thus, kinetic stability involves the particle’s own movement (Brownian motion), which increases the repulsion between the droplets in order to provide stability to the nanoemulsion, unlike the thermodynamic stability that involves the chemical balance of the different constituents of the system, which leads to a reduced state of energy. This kinetic stability offers the system an extended resistance against the processes of creaming, sedimentation and flocculation, even when subjected to temperature changes, in contrast to the thermodynamic stability of microemulsions [61,74]. This effect is demonstrated by Stokes’ law (Equation (1)), which shows that the creaming rate decreases with the decrease in the dimensions of the internal phase [67].

(1) $$v=\frac{d^{2}\left(p-p_{0}\right)g}{18_{\eta }}$$

where v is the speed of the formation of creaming; *d* is the internal diameter of the droplet; *p* and *p*_0_ are the densities of the internal phase and the dispersion medium, respectively; *g* is the gravity constant; η is the viscosity of the dispersion medium. Characteristics such as the kinetic stability of nanoemulsions, as well as their appearance, are, thus, due to the small dimensions of the droplets that compose these systems [76]. At the same time, the good stability of nanoemulsions avoiding the aggregation of droplets could also be related to interaction forces. Interaction forces are related to the surface charge of the droplet, which cause them to maintain an equidistance from each other, similar to a network. This attractive force that exists between the droplets reduces with the size of the particles [20,69,70]. The Brownian movement, typical of some pharmacological systems, promotes a flow of diffusion through the skin [77]. Taking this effect into account, and since nanoemulsions have the main characteristic of an intense Brownian movement, this type of system should provide better penetration through the skin due to its kinetic activity.

This type of formulation is widely used in drugs that are poorly soluble in water and in lipids [78]. The advantages provided by nanoemulsions include (i) the easy solubilization of drugs, (ii) the increase in therapeutic efficacy and, consequently, (iii) the reduction in side effects [79,80].

The use of nanoemulsions to enhance the solubility of drugs for topical application has been demonstrated and confirmed through several studies. An example is prednicarbate, a next-generation corticosteroid with a good benefit/risk ratio, but with a reduced water solubility. Nanoemulsions containing prednicarbate promote an improvement in penetration and in the drug delivery capacity of this corticosteroid, being advantageous for the treatment of atopic dermatitis [81]. The anticancer potential of caffeine has recently been discovered, so there is interest in its topical application for skin cancer treatment. Nanoemulsions are interesting delivery systems for caffeine [82], suggesting that this drug may have an improved transdermal penetration when formulated in nanoemulsions.

In contrast to microemulsions, nanoemulsions generally require more energy to be produced [23]. To produce nanoemulsions, a disruptive force is used to supplant the necessary free energy, breaking the oily phase in order to increase the area between the two phases so that they are dispersed in the form of thin, homogeneous droplets [23,83,84]. For this purpose, high and low-energy methods are available. In high-energy methods, the pre-emulsion formed by mechanical dispersion (Ultra-turrax^®^, IKA^®^-Werke GmbH & Co. KG, Staufen, Germany) is subjected to emulsification forces, i.e., by means of high-pressure homogenization (HPH) or by ultrasound [23,59,75,84,85,86,87]. Low-energy methods include minimal energy expenditure, i.e., spontaneous emulsification [85,86].

This set of techniques generates intense and disruptive forces that lead to a mechanical breakdown of the lipophilic phase in small droplets that are dispersed in the hydrophilic phase [18]. The droplet size depends on the method applied and the operating conditions (time and temperature), as well as the composition of the system [84].

In classical methods, when preparing a nanoemulsion, it is necessary to formulate a pre-emulsion, in which all the constituents are mixed. In this mixture, rotor forces (i.e., ultra-turrax) are applied, which promote an initial dispersion. However, they are not very effective, since they do not have a good dispersion capacity in terms of droplet size. In fact, the energy provided by these devices mostly dissipates in heat and friction, so they need complementary devices (i.e., HPH) to provide additional free energy sufficient to create an adequate interphase contact area [61]. HPHs are designed to force the passage of a pre-emulsion through narrow spaces by means of high pressure. The formulation undergoes a sudden acceleration, reaching speeds close to 300 m·s^−1^. As a result, small volumes of formulation are subjected to cutting forces, impact and cavitation, which create droplets at the nanoscale [61].

Lately, some relevance has been given to the ultrasound process, due to the reduced energy consumption, the reduction in the amount of surfactant, and the obtainment of particles of even smaller sizes when compared to the methods previously described [23]. Its operation has been described previously. However, in the case of sensitive pharmacologically active compounds (such as peptides, proteins or nucleic acids), high-energy methods can lead to degradation, denaturation or the loss of SFA activity throughout the process [61]. For this purpose, there are alternatives, such as low-energy processes, for the formation of these emulsions.

The reduced energy methods make use of the remaining force in the different components of the nanoemulsions [67]. These methods make use of the physico-chemical properties of the system, only requiring gentle agitation, leading to a cost reduction inherent to its production [75]. The nontoxic and nonirritating properties, conferred by the proportions and nature of its components, make these systems ideal therapeutic agents, so they do not damage human and animal cells [84].

Table 1 summarizes recent advances concerning the use of micro- and nanoemulsions as delivery systems for both synthetic and natural drugs. As observed, the development of new products for the pharmaceutical and cosmetic industries that retain or improve their bioactivity while presenting a higher bioavailability are the main source of new scientific publications regarding the use of micro and nano-scaled emulsions.

Pharmaceutical products already well established in the market for topical application, such as, for example, ibuprofen, 5-fluorouracil, coenzyme Q10 or clotrimazole, are frequently attractive for these studies, since the development of a more effective formulation with a lower dose of the drug is of high interest. For example, regarding ibuprofen, formulations containing natural lipophilic phases, which can improve its biocompatibility and decrease the toxicity (as observed for some commonly used surfactants, such as CTAB [88] or synthetic surfactants (Tween^®^ 20)), have been shown to increase the drug’s solubility and permeability, while presenting the anti-inflammatory and analgesic activity. Similar results were reported for 5-fluorouacil and clotrimazole, whose formulations increased the efficacy while exhibiting antiproliferative and antifungal activities, respectively.

**Table 1:** Microemulsion and nanoemulsion technology applications to improve natural and synthetic drug delivery and bioactivity.

Drug	Formulation	Physical Characterization	Experimental Model	Bioactivity/Effect	Ref
Ibuprofen	o/w Palm olein ester	MPS: 20.90 nm PDI: n.s. ZP: n.s.	Rat	Anti-inflammatory Analgesic activity Increased drug solubility Increased permeability	[89]
	o/w Menthol	n.s.			
	o/w Limonene	n.s.			
	o/w Ethyl oleate, Tween^®^ 20, ethanol	MPS: 46 nm PDI: n.s. ZP: n.s.	Rat	Increase permeability Anti-inflammatory activity	[90]
Pioglitazone	o/w Castor Oil, Labrasol^®^, propylene glycol	MPS: 182 nm PDI: 0.352 ZP: 12.37 mV	Human skin Mice	Increase permeability Anti-inflammatory activity	[91]
Retinyl palmitate	o/w Labrafac^®^ lipophile, Labrasol^®^, Plurol^®^ oleique	MPS: 14.42 nm PDI: 0.68 ZP: n.s.	Human skin	Increased permeability	[92]
Capsaicin	o/w Olive oil, Tween^®^ 80, Span^®^ 80, ethanol	MPS: 13.20 nm PDI: 0.68 ZP: 2.80 mV	Rat Rabbit	Anti-inflammatory Analgesic	[93]
Betulin	o/w Flax-seed oil, egg phosphatydilcholine	MPS: n.s. PDI: n.s. ZP: −23 mV	Mice	Increase solubility and bioavailability Anti-inflammatory activity Anticarcinogenic activity	[94]
Eugenol	o/w Eugenol, Tween^®^ 80, Labrasol^®^	MPS: 89.98 nm PDI: 0.006 ZP: −10.05 mV	Rat	Anti-inflammatory activity	[95]
	o/w Eugenol, Tween^®^ 20, isopropyl alcohol	MPS: 24.40 nm PDI: 0.30 ZP: 0.33 mV	Rat	Anti-inflammatory activity	[96]
Etoricoxib	o/w Triacetin, Cremophor^®^ RH 40, Transcutol^®^ P	MPS: 50.67 nm PDI: 0.44 ZP: n.s.	Porcine skin	Increased drug delivery Anti-inflammatory activity	[97]
Mangiferin	o/w Lipoid, polysorbate 80, tocopherol, almond oil, hyaluronic acid	MPS: 195.50 nm PDI: 0.21 ZP: −38.02 mV	Porcine skin Mice	Increased permeability Anti-inflammatory activity	[98]
Clobetasol propionate	o/w Eucalyptus oil, Tween^®^ 20, ethanol	n.s.	Rat	Anti-inflammatory activity	[99]
	o/w Algal oil, Tween^®^ 20, PEG 200	MPS: 120 nm PDI: 0.33 ZP: −37.01 mV	Rat	Increased permeability Anti-inflammatory activity	[100]
β-Caryophyllene	o/w Copaiba oil, Span^®^ 80, Tween^®^ 20 or CTAB	MPS: 223.67 nm PDI: 0.18 ZP: 34.57 mV	Mice	Anti-inflammatory activity	[101]
Curcumin	o/w Clove oil, Tween^®^ 80, PEG 400	MPS: 93.64 nm PDI: 0.26 ZP: −11.67 mV	Rat	Increased permeability Wound healing capacity Anti-inflammatory activity	[102]
	o/w Limonene, lecithin, ethanol,	MPS: 20 nm PDI: 0.1 ZP: −0.1 mV	Human skin	Increased permeability	[103]
	o/w Eucalyptol, lecithin, ethanol,	MPS: 15 nm PDI: 0.10 ZP: 0 mV			
Tacrolimus Omega 3	o/w Fish oil, Tween^®^ 80, Transcutol P	MPS: 116.30 nm PDI: 0.18 ZP: −3.99 mV	Mice	Psoriasis treatment	[104]
	o/w Linseed oil, Tween^®^ 80, Transcutol P	MPS: 126.30 nm PDI: 0.19 ZP: −3.13 mV			
Methotrexate Resveratrol	o/w Acrysol K^®^, Tween^®^ 20, Transcutol P^®^	MPS: 55.43 nm PDI: n.s. ZP: −26 mV	Rat	Anti-inflammatory activity Antiarthritic activity	[105]
Clotrimazole	o/w Labrafac^®^ lipophile, Labrasol^®^, capryol	MPS: 186 nm PDI: 0.41 ZP: −5.61 mV	Human skin	Antifungal activity	[106]
Fullerene	o/w Palm kernel oil, Span^®^ 80, Tween^®^ 80	MPS: 175 nm PDI: n.s. ZP: n.s.	Human fibroblast cell line (3T3)	Prevent collagen degradation and dehydration Antiaging activity	[107]
α-Lipoic acid	w/o and o/w Shea butter, squalane, stearol, Plurol^®^ oleique CC497, Maisine^®^ 35-1	MPS: 5.08 µm PDI: n.s. ZP: n.s.	Rat	Antiaging activity	[108]
Quercetin	o/w Peppermint oil, Cremophor EL^®^, 1,2-propanediol	MPS: 12.66 nm PDI: 0.27 ZP: −4.35 mV	Rat	Improved solubility and skin permeation Antiaging activity	[109]
	o/w Clove oil, Cremophor EL^®^, 1,2-propanediol	MPS: 9.74 nm PDI: 0.08 ZP: −0.99 mV			
	o/w Rosemary oil, Cremophor EL^®^, 1,2-propanediol	MPS: 11.61 nm PDI: 0.39 ZP: −4.05 mV			
	o/w Arachis oil, oleic acid, Tween^®^ 20, PEG-400	MPS: 136.8 nm PDI: 0.27 ZP: −25.4 mV	Rat	Increased permeability Antiarthritic activity	[110]
Coenzyme Q10	o/w Isopropyl myristate, Cremophor^®^, Transcutol^®^	MPS: 16.89 nm PDI: 0.04 ZP: −13.1 mV	HaCaT, human keratinocytes cell line NIH3T3, human fibroblasts cell line	Wound-healing activity Improved solubility and skin permeation	[111]
	o/w Isopropyl myristate, Tween^®^ 80, Transcutol^®^ HP	MPS: 94. 60 nm PDI: n.s. ZP: −18 mV	Rat	Increased solubility and permeability Antiwrinkle activity	[112]
5-Fluorouracil	o/w Lauroglycol-90, Transcutol^®^ HP, isopropyl alcohol	MPS: 68.20 nm PDI: 0.22 ZP: −25.92 mV	SK-MEL-5 cell line	Chemopreventive activity	[113]
Pentoxifylline	w/o Caprylic/capric triglycerides, Tween^®^ 80, Brij 52	MPS: 67.36 nm PDI: n.s. ZP: n.s.	Rat	Anti-inflammatory activity	[114]
Naringenin	o/w Wheat germ oil, oleic acid, Cremophor^®^ EL, Tween^®^ 20	MPS: 249.05 nm PDI: 0.41 ZP: n.s.	A431 cell lineRat	Antioxidant activity Decreased photoaging Anticancer activity	[115]
Ferulic acid	o/w Isostearyl isostearate, labrasol, plurol isostearique	MPS: 102.3 nm PDI: 0.16 ZP: −35.20 mV	Rat	Increased permeability Protection against UV radiation	[116]
Octylmethoxycinnamate, octocrylene, diethylamino hydroxybenzoyl hexyl benzoate, benzophenone-3, pomegranate extract	o/w Chitosan, Tween^®^ 80, Span^®^ 80	MPS: 109 nm PDI: n.s. ZP: n.s.	Rat	Increased skin retention Photoprotection	[117]
Resveratrol	o/w Sefsol 218^®^, PEG 400, Tween^®^ 80	MPS: 50.04 nm PDI: 0.17 ZP: n.s.	Rat	Protection against UV radiation Antioxidant activity	[118]

Notes: Mean particle size (MPS); zeta potential (ZP); not specified (n.s.).

Additionally, drugs from natural sources have gained new interest when combined with micro and nanoemulsions as delivery systems. Phytochemicals with a low water solubility can be effectively transported across the skin, increasing its bioavailability, as is the case of quercetin. The inclusion of this flavonoid in microemulsions proved to be an effective formulation to enhance its antiaging effect [109]. In the same way, but using a nanoemulsion, an increased quercetin permeation contributed to the development of a promising formulation for arthritis treatment [110].

Concerning wound-healing activity, products highly sought after for topical application, a coenzyme Q10 microemulsion [111] and a curcumin nanoemulsion [102], have been reported as potential candidates for creams for wound treatment, while through the use of phytochemicals such as resveratrol [118] or ferulic acid [116], nanoemulsion-based gels have been developed as new alternatives for UV protection products.

As seen in Table 1, the wide range of natural and synthetic drugs with described pharmaceutical potential could benefit from being included in emulsion-based delivery systems, which creates endless possibilities for new solutions for medical applications and new products for the highly demanding cosmetic industry. Several products based on emulsions are already available for generic consumption, mainly consisting of creams for the topical application of anti-inflammatory drugs (e.g., diclofenac; Voltaren Emulgel, GSK, Brentford, UK), burnt skin treatment (e.g., trolamine; Biafine, Johnson & Johnson) or more frequently as antiaging products produced by brands such as Mibelle, Avène or Vichy. Table 2 summarizes some of the products available in the market that are formulated as emulsions.

As seen in Table 2, there are several products already commercialized using both natural and synthetic drugs formulated as emulsions. Standing out among the various applications, the cosmetic industry is the major supplier of emulsion-based products, where antiaging, cleansing, moisturizing or antiwrinkle creams represent the vast majority of consumer demand.

## 5. SolEmul^®^ Technique

Approximately 60% of molecules developed, mainly by synthesis, have a solubility of less than 0.1 mg/mL, either in hydrophilic or in lipophilic media [73,78,119,120,121,122,123]. As a result, these have a low bioavailability [78]. In fact, there are some methods to deliver problematic molecules that include (i) the use of solvent mixtures, (ii) surfactant-promoting surfactants, (iii) a mixture of surfactants and/or solvents, (iv) a mixture of micelles, (v) o/w emulsions and (vi) the use of organic solvents [78,119,124,125]. These methods involve packaging the molecule in nanoparticle systems (e.g., polymeric nanoparticles) and lipid systems (i.e., o/w emulsions and liposomes) [73,119]. However, all of these processes have shown some limitations for drug targeting, even presenting cases of toxicity or irritation at the application site [125]. However, studies developed by Müller in the last decade, brought a new technique called SolEmul^®^ technology that can be applied to all types of molecules with solubility problems [73,78,79,119,120,124,125,126].

Thus, the SolEmul^®^ technique was created to overcome the barrier of a poor solubility, high production costs and the associated high toxicity, and can be performed in two ways [120,126]. The first is based on mixing drugs in a preformed emulsion available on the market (i.e., Lipuro^®^, Intralipid^®^ and Lipofundin^®^) [120,124,127,128]. The resulting mixture is homogenized until the drug crystals are completely dissolved [127]. The second method is the production of an emulsion, in which the drug is dissolved in the droplet interphase [119,120,124,126].

Both processes result in an ultrafine nanoemulsion, in which the drugs are stabilized by lecithin (surfactant) or a mixture of surfactants (lecithin and another surfactant), and the drugs are located at the system interface [119,124,126]. The dissolution process can be accelerated by high-speed mechanical stirring methods (i.e., Ultra-turrax^®^) [119,120,124]. From here, a pre-emulsion can be obtained, which is processed by HPH to reduce the inner droplet size and the placement of the drug at the interface [119,120,128]. This process can be carried out by applying 1 to 20 homogenization cycles, with a pressure of about 1500 bar [119,120,124,126]. The formulation obtained can be colorless or translucent, depending on its composition.

However, there are some limitations to the packaging of drugs in o/w nanoemulsions. It should be noted that the interface is saturated with drugs, and the presence of small undissolved crystals in the dispersion is inevitable. To overcome this problem, it is essential to choose a concentration of drug lower than its saturation concentration [119,126].

## 6. Supersaturated Self-Nanoemulsified Drug Delivery Systems

Supersaturated self-nanoemulsified drug delivery systems comprise an isotropic mixture of oil, water, surfactant and cosurfactant [129]. These systems consist of self-emulsified drug distribution systems, with a high thermodynamic stability and polymers in their composition, whose function is to inhibit the precipitation process of the excess active substance in order to maintain a supersaturated solution of the temporary drugs after dispersion; thus, allowing the absorption [130]. These systems, therefore, have a high solubilization capacity, in addition to an excellent stability [129].

They are prepared by incorporating powders into liquid excipients by solidification, giving rise to o/w nanoemulsions smaller than 200 nm after gentle agitation in an aqueous medium. These fine nanoemulsion droplets have the advantage of having dissolved drugs with a high interface surface area, which increases the absorption of the molecule, enabling a uniform and reproducible bioavailability [129,130,131].

This method provides the spontaneous formation of particles with dimensions ranging from 20 to 200 nm [132,133]. Supersaturated self-nanoemulsified drugs are becoming a viable way to solve problems related to the solubility of poorly water-soluble molecules [134]. In comparison with other emulsions, this type of formulation can be considered a sensitive dispersion, with physical stability, being metastable and achievable with a simple procedure, which can offer an improvement in the dissolution and absorption rates of drugs [132]. As they are supersaturated systems of drugs, they enable an increase in the thermodynamic activity of the molecule, which has led to them being extensively studied for topical use [130].

Table 1 and Table 2 summarized recent advances concerning the use of micro and nanoemulsions as delivery systems for both synthetic and natural drugs. As observed, the development of new products for the pharmaceutical and cosmetic industries, that retain or improve their bioactivity while presenting higher bioavailability, is the main source of new scientific publications regarding the use of micro and nano-scaled emulsions. Nevertheless, this delivery system still faces some limitations. In order to produce more microemulsion- and nanoemulsion-based products based on recent advancements in production and oil-phase compositions mentioned above, extensive studies should be performed regarding the toxicity of the components, the efficacy of the product and production cost. An additional concern is related to the maintenance of the product’s attractiveness to consumers, as modifications towards a less toxic formulation may induce less appealing or less stable formulations that reduce the product’s viability. Adding to the challenges, new innovations in micro- and nano-sized emulsions need to undergo a scale-up process in order to be mass produced, where the compatibility of the ingredients is a challenging process. Finally, the environmental fate of the formulations should also be addressed [135,136].

## 7. Conclusions

The skin is definitely a very promising route for drug administration and delivery. The ability to release drugs into the bloodstream at constant concentration levels, coupled with its high application area, the exemption of the first-pass effect and application comfort, make it the route of choice. Both microemulsions and nanoemulsions are carriers able to act as permeation/penetration enhancers through the skin due to the characteristics they bring together, allowing to overcome the barrier of the *Stratum corneum*. They may also increase the liposolubility of hydrophilic drugs, because a more nonpolar chain can be added to its more polar side, which gives ambivalence to molecules with bioavailability problems. The same happens with lipophilic substances, so that their more nonpolar side is added to a more polar chain. Microemulsions and nanoemulsions have similar macroscopic characteristics. Although they are microemulsions, they have a droplet size smaller than that of nanoemulsions, which can cause some confusion. However, what distinguishes nanoemulsions is their kinetic behavior, conferred by the Brownian motion of the particle. Microemulsions have a high thermodynamic stability, making their shelf life longer compared to classic emulsions. On the other hand, despite having unstable thermodynamics, nanoemulsions have kinetic stability, which makes them resistant to degradation problems. These characteristics make these systems advantageous in the preservation of drugs, so that they remain in good condition until the moment of application. The high thermodynamic stability, as well as the kinetics, make these systems able to increase the permeation capacity in the skin and the drug’s sharing quotient, which promotes a good ability to overcome transdermal barriers. Both the behavior of these systems and the components of which they are made make these systems great promoters of skin permeation, which makes them good vehicles for transdermal drug delivery. The extensive study of these systems gave rise to a set of production methods, with different characteristics chosen according to the availability of the laboratory and the properties of the molecules to be transmitted. It should be noted that in the case of nanoemulsions, it is possible to produce systems capable of successfully increasing the bioavailability of drugs that are poorly soluble in hydrophilic and lipophilic media, which can be a good solution to the problems faced by many drugs on the market. The existence of spontaneous production methods is of great value, especially in periods of resource containment, so there is a significant reduction in energy costs as well as high-cost machinery. These methods can be very advantageous in the case of large-scale production. However, it would be important to conduct further comparative studies between microemulsion and nanoemulsions system, in order to distinguish them unequivocally, which is the best method for application to the skin. This gap is due to the fact that they are still very novel in the scientific world.

## Figures and Tables

**Table 2:** Examples of pharmaceutic and cosmetic products, available on the market, formulated as emulsions.

	Drugs	Skin Cream	Application
Nanoemulsion	Lipophilic fraction of cocoa beans	NanoCacao Mibelle Biochemistry	Antiaging
	Coenzyme Q10 Tocopherol Vitamin C derivative	Nano-Lipobelle™ DN CoQ10 oA Mibelle Biochemistry	Collagen production Protects against photoaging Antiaging
	Omegas 3, 6, 7 and 9	NanoVit oA Mibelle Biochemistry	Antiaging Regenerate Photo protection
	Coenzyme Q10 Vitamin C and E	NanoMax Mibelle Biochemistry	Antiaging Antioxidant
Emulsion	Vitamin B3	SkinCeuticals Metacell Renewal B3 Serum	Protects against photoaging Antiwrinkle Antiaging
	Bamboo extract Green tea extract	Thera Emulsion DR ORACLE	Antiacne Moisturizer
	Hyaluronic Acid Urea	Hyluronic Urea Emulsion Dalton	Moisturizer Antiaging Antiwrinkle
	Jojoba Oil	Jordan Dead Sea Salt Moisturizing Emulsion	Moisturizer Against skin rash
	Crambe Maritima	S.E.A. EMULSION Marine Stem Cell System Dalton	Moisturizer Antiaging Antiwrinkle Antioxidant
	Trolamine	Biafine	Wound healing Burnt skin and solar erythema treatment
	Diclofenac	Voltaren Emugel	Anti-inflammatory Analgesic
	APG Gluco-glycerol Hyaluronic acid	Eucerin DermatoCLEAN Cleansing Milk	Moisturizer
	Zinc oxide Vitamin E	Isdin Phoyo Eryfotona Actinica	Solar protection Antioxidant
	Coenzyme Q10	Lightweight Q10 Anti-Aging Moisturizer to Boost Cell Activity—DALTON	Moisturizer Antiwrinkle Antioxidant
	Not specified	Hydrance LIGHT Hydrating Emulsion Avène	Moisturizer
	Not specified	PhysioLift DAY Smoothing Emulsion Avène	Antiaging Antiwrinkle Antioxidant
	Not specified	Cicalfate Post-Procedure Emulsion Avène	Moisturizer Wound Care
	Not specified	Tolérance Extrême Emulsion Avène	Moisturizer
	Not specified	Normaderm Phytosolution VICHY	Skin cleanser
	Not specified	Capital Soleil Dry Touch SPF 50 VICHY	Photoaging

## Data Availability

Not applicable.
